# A national study of firearm exposure and safety training among rural youth

**DOI:** 10.1186/s40621-024-00533-1

**Published:** 2024-09-05

**Authors:** Jamie L. Koopman, Benjamin M. Linden, Megan R. Sinik, Kristel M. Wetjen, Pam J. Hoogerwerf, Junlin Liao, Charles A. Jennissen

**Affiliations:** 1 Department of Emergency Medicine, Franciscan Health-Olympia Fields, 20201 Crawford Ave, Olympia Fields, Illinois, 60461 USA; 2 Department of General Surgery, University of Minnesota Medical School, University of Minnesota, 500 SE Harvard St, Minneapolis, MN 55455 USA; 3 Department of Internal Medicine, Beth Israel Deaconess Medical Center, Harvard Medical School - Harvard University, 330 Brookline Ave, Boston, MA 02415 USA; 4 Division of Pediatric Surgery, Department of Surgery, University of Iowa Health Care Stead Family Children’s Hospital, 200 Hawkins Drive, Iowa City, IA 52242 USA; 5 Injury Prevention and Community Outreach Program, University of Iowa Health Care Stead Family Children’s Hospital, 200 Hawkins Drive, Iowa City, IA 52242 USA; 6 Department of Surgery, Roy J. and Lucille A. Carver College of Medicine, University of Iowa, 200 Hawkins Drive, Iowa City, IA 52242 USA; 7 Department of Emergency Medicine, Roy J. and Lucille A. Carver College of Medicine, University of Iowa, Iowa City, USA; 8 Stead Family Department of Pediatrics, Roy J. and Lucille A. Carver College of Medicine, University of Iowa, Iowa City, USA

**Keywords:** Adolescent, Farms, Firearm, Handgun, Hunting, Rifle, Rural, Shooting, Shotgun, Training, Youth

## Abstract

**Background:**

Data regarding rural youths’ experience with firearms, including safety training, is highly limited despite their frequent presence in homes. Our objective was to investigate rural adolescents’ use of firearms and whether they had received formal firearm training.

**Methods:**

A convenience sample of 2021 National FFA (formerly Future Farmers of America) Convention & Expo attendees were given an anonymous survey at the University of Iowa Stead Family Children’s Hospital injury prevention booth. The survey explored their use of rifles/shotguns and handguns and whether they had completed a certified firearm safety course. Descriptive and comparative analyses, including multivariable logistic regression analyses, were performed on compiled data.

**Results:**

3206 adolescents ages 13–18 years participated with 45% reporting they lived on a farm or ranch. The vast majority of participants (85%) had fired a rifle/shotgun; 43% reported firing them > 100 times. Of those that had fired rifles/shotguns, 41% had done so before 9 years old. Most had also fired a handgun (69%), with 23% having fired handguns > 100 times. Of those that had fired handguns, 44% had done so before 11 years. Average age for first firing rifles/shotguns was 9.5 (SD 3.1) years, and 11.1 (SD 3.0) years for handguns. Males, non-Hispanic Whites, and those living on farms or in the country had significantly greater percentages who had fired a rifle/shotgun or a handgun. Significant differences were also seen by U.S. census region. Over half (64%) reported having gone hunting. Of those that had used a firearm, 67% had completed a firearm safety training course. Overall, 23% were/had been members of a school or club shooting team and of these, 87% had taken a safety course.

**Conclusions:**

Most FFA member participants had fired both rifles/shotguns and handguns, many at very young ages. Significant differences in firearm use were noted by demographic factors including the home setting (i.e., farms and ranches) and their U.S. census region. Nearly one-third of adolescent firearm users had not received formal safety training. Promoting firearm safety should include advising families on when it is developmentally appropriate to introduce youth to firearms and on the importance of firearm safety training.

## Background

Firearm injuries in children and adolescents are a rising concern across the U.S. and have become the leading cause of death for those 19 years of age and younger (Goldstick et al. 2022). According to the Centers for Disease Control and Prevention (CDC), there were 18,800 non-fatal injuries and 2601 deaths in youth under 18 years due to firearms in 2021 (Centers for Disease Control and Prevention 2021). This was a 50% increase in firearm mortality as compared to 2019 (Gramlich 2021). In the U.S., more than 40% of households with children have firearms with 15% of those households storing them loaded and unlocked, potentially allowing anyone within the home access (Miller and Azrael 2024). In addition, in households with children and firearms, nearly two-thirds do not store the firearms both locked and unloaded which means approximately 16 million U.S. children live in a household with a firearm unsafely stored (Miller and Azrael 2024; Azrael et al. 2015).

Firearm ownership in the U.S. is higher than any other country (Gun Ownership By Country 2024; Karp 2018), and the proportion of homes with firearms in rural settings is significantly greater than urban communities (Sadowski and Munoz 1996; Senturia et al. 1994; Shaughnessy et al. 1999; Smith 2001; Azrael et al. 2017; Nordstrom et al. 2001; Jennissen et al. 2021). Studies have also found a higher prevalence of handgun carrying among rural teenagers than their urban counterparts (Rowhani-Rahbar et al. 2020; Substance Abuse and Mental Health Services Administration 2023). While the number and rates of homicide deaths due to firearms are much greater in urban settings, rural areas have higher rates of both firearm-related suicides and unintentional deaths (Branas et al. 2004; Fontanella et al. 2015; Nestadt et al. 2017). As for pediatric firearm-related injuries, the hospitalization rate for those under 15 years is higher for rural than urban youth with unintentional injuries being the most common cause (Herrin et al. 2018).

We previously surveyed nearly 1400 rural adolescents who were attendees of a state FFA conference in Iowa (Miller et al. 2024). FFA is a national organization associated with schools that focuses on agricultural education and leadership development. The vast majority (85%) of respondents had fired a rifle/shotgun and over three-fifths (62%) had fired a handgun. Many used firearms frequently and they had started at very young ages. Of those that had used a firearm, only about 60% had completed a firearm safety certification course.

With the recent dramatic rise in pediatric firearm-related injuries and deaths, there has been an increase in firearm-related research (Miller and Azrael 2024; Jennissen et al. 2021), but few studies that have focused on the use of firearms by youth. Given that our previous study was limited to the state of Iowa, we wanted to determine firearm use using a national sampling of rural adolescents. Our study’s objectives were to explore rural adolescents’ use of firearms and whether they had completed a certified firearm safety course. We also wanted to determine if there were differences by demographic factors including the region of the country where youth lived.

## Methods

### Study population

A cross-sectional survey study involving a convenience sample of attendees at the 2021 National FFA Convention & Exposition in Indianapolis, Indiana, was performed at the University of Iowa Stead Family Children’s Hospital injury prevention booth. As of 2024, there were nearly 950,000 FFA members in over 9000 local chapters across all 50 states, Puerto Rico and the U.S. Virgin Islands (National FFA Organization 2024). FFA members are in grades 5–12 and there are some collegiate chapters as well. Conference attendees were recruited to complete the anonymous survey on paper or by cell phone linking via a QR code to an electronic survey on Qualtrics (Qualtrics International, Inc, Provo, UT). Staff reviewed the written surveys for completeness. Participants received a modest prize (e.g., lip balm, trucker hat) determined via Plinko board as an incentive to complete the survey. Study inclusion was restricted to English speakers 13–18 years old.

### Survey

The survey was created by the members of the University of Iowa Stead Family Children’s Hospital’s Injury Prevention Program, along with staff and students interested in firearm injury prevention at the study institution. The written survey was administered to 20 young persons aged 11–22 years for validation. After completion, these individuals were verbally asked for their input and clarification of their responses for any survey questions not easily understood. All responses provided from the validation were compared for consistency and were utilized in improving the survey’s final design. After being used for the state Iowa FFA conference (Jennissen et al. 2021; Miller et al. 2024; Jennissen et al. 2021), the survey was modified slightly for the national conference.

Demographic variables included age (years), gender (female, male, other, choose not to respond), race/ethnicity (Asian, Black/African American, Hispanic/Latinx, Native American/Alaska, Native Hawaiian/Pacific Islander, White/Caucasian, Mixed, Other), where they lived (on a farm, in the country/not on a farm, in town), and the US state of residence. Participants could select all that apply for race/ethnicity. In the questionnaire, members were asked how many separate occasions they had fired rifles/shotguns and handguns. Response choices included: Never, < 10 times, 10–100 times, > 100 times. If they had fired a rifle/shotgun or a handgun, they were asked to specify at what age in years they had first fired one.

Participating individuals were asked if they had ever gone hunting and, if so, at what age they first went hunting with a rifle/shotgun. They were asked whether they had ever been a member of a school or club shooting team. In addition, participants were queried whether they had ever taken a formal/certified hunter or firearm safety training course and, if so, at what age had they first completed it.

### Data analysis

Written and electronic surveys were provided to the research team. The Institutional Review Board regarded the study exempt as the analysis was completed on an existing, anonymously gathered dataset. The written surveys were inputted into Qualtrics™ with those of participants who had completed the survey by cell phone. Aggregated data was exported via Excel (Microsoft Corp, Redmond, Washington) and imported into Stata 15.1 (StataCorp, College Station, Texas).

Descriptive (frequencies), bivariate (chi-square, Fisher’s exact test) and multivariable logistic regression analyses were performed. Sixteen respondents (0.5%) noted their gender as “other” and were not included in the comparative analysis regarding sex. The race/ethnicity variable was divided into “non-Hispanic (NH) White” and “other races/ethnicities” due to the limited diversity in the participant population. This resulted in significant heterogeneity within the other races/ethnicities group; however, it allowed for use of the variable in the data analysis. The states in which the participants lived were grouped by U.S. census region which are West, South, Midwest and Northeast. All *p*-values were two-tailed and a value < 0.05 was considered statistically significant. Fisher’s exact test was utilized for any comparison in which a cell had a predicted value of < 5. Missing data were not included in analyses.

## Results

### Subject demographics

Completed surveys were obtained from 3296 adolescents (13–18 years old). See Table 1. The percentage of males and females was similar and about three-quarters of participants were 15–17 years old. Nearly half lived on a farm, one-third lived in the country/not on a farm, and one-fifth lived in town. Ninety-two percent were NH White. Participants were from Puerto Rico and every U.S. state except Maine, New Hampshire, Vermont and Massachusetts. Two-thirds of participants were from the Midwest, just over one-fifth were from the South, and 9% and 3% were from the West and Northeast U.S. census regions, respectively.

**Table 1:** Demographics, firearm use and safety training among adolescent survey respondents at the 2021 National FFA Convention & Expo

	n (Col %)^a^
Group N	3296
*Sex*	
Male	1623 (49)
Female	1639 (50)
Other	16 (< 1)
*Age*	
13 years	60 (2)
14 years	327 (10)
15 years	710 (22)
16 years	890 (27)
17 years	947 (29)
18 years	353 (11)
*Residence*	
Farm	1495 (45)
Country/not farm	1116 (34)
Town	679 (21)
*Race/ethnicity*	
Non-Hispanic White	3025 (92)
Other races/ethnicities	261 (8)
*US census region*	
Midwest	2173 (66)
South	692 (21)
Northeast	111 (3)
West	294 (9)
*Fired a rifle/shotgun*	
> 100 times	1411 (43)
10–100 times	873 (27)
< 10 times	502 (15)
Never	401 (15)
*Fired a Handgun*	
> 100 times	736 (23)
10–100 times	845 (26)
< 10 times	659 (20)
Never	1014 (31)
*Have been hunting*	
Yes	2089 (64)
No	1161 (36)
*School/club shooting team member*	
Yes	730 (23)
No	2491 (77)
*Firearm safety training*	
Yes	1945 (60)
No	1321 (40)

^a^The sum of n may not equal the total Group N due to missing values

### Firearm use and safety training

Eighty-five percent of participants reported having fired a rifle/shotgun, with over 40% reporting having fired one > 100 times. See Table 1. Additionally, over two-thirds reported having fired a handgun; approximately one-fifth had done so > 100 times. Nearly two-thirds of respondents reported having gone hunting with firearms, and more than one fifth stated they were or had been a member of a school or club shooting team. Overall, six in ten reported having completed a certified firearm safety training course.

### Comparisons of rifle/shotgun use

Among those who had fired a rifle/shotgun, almost half (1244/2657, 47%) had done so before they were 10 years old and over four-fifths (2198/2657, 83%) before 13 years. The average age participants had first fired a rifle/shotgun was 9.4 years (SD 3.1 years).

Males as compared to females, those living on farms or in the country/not on a farm as compared to those from towns, and NH Whites as compared to other races/ethnicities all had greater proportions that had fired a rifle/shotgun. See Table 2. Those from the West, South and Midwest U.S. census regions had similar but higher percentages than those from the Northeast.

**Table 2:** Demographic comparisons of rifle/shotgun use among survey respondents at the 2021 National FFA Convention & Expo

Variables	Cross tab analysis			Logistic regression analysis^a^	
	Yes	No	*p* value	OR	95% CI
	n (Row %)^b^	n (Row %)^b^			
**Fired a rifle/shotgun**^c^					
*Sex*					
Male	1513 (94)	99 (6)	< 0.001	4.52	3.55–5.75
Female	1249 (77)	382 (23)		1.0 (ref)	
* Age*					
16–18 years	1849 (85)	329 (15)	0.695	1.04	0.83–1.30
13–15 years	931 (85)	159 (15)		1.0 (ref)	
*Residence*					
Farm	1341 (90)	145 (10)	< 0.001	3.44	2.67–4.45
Country/not farm	959 (86)	151 (14)		2.44	1.89–3.16
Town	483 (72)	192 (28)		1.0 (ref)	
*Race/ethnicity*					
Non-Hispanic White	2597 (86)	412 (14)	< 0.001	2.5	1.82–3.45
Other races/ethnicities	181 (70)	78 (30)		1.0 (ref)	
*U.S. census region*					
Midwest	1835 (85)	324 (15)	0.003	1.97	1.23–3.15
South	591 (86)	99 (14)		2.37	1.42–3.95
West	258 (88)	34 (12)		3.24	1.78–5.91
Northeast	82 (74)	29 (26)		1.0 (ref)	
**Frequent firing of rifles/shotguns (defined as > 100 times)**^d^					
* Sex*					
Male	1011 (63)	601 (37)	< 0.001	5.32	4.54–6.22
Female	396 (24)	1235 (76)		1.0 (ref)	
*Age*					
16–18 years	962 (44)	1216 (56)	0.085	1.24	1.05–1.47
13–15 years	447 (41)	643 (59)		1.0 (ref)	
*Residence*					
Farm	768 (52)	718 (48)	< 0.001	2.84	2.29–3.53
Country/not farm	460 (41)	650 (59)		1.90	1.51–2.38
Town	182 (27)	493 (73)		1.0 (ref)	
*Race/ethnicity*					
Non-Hispanic White	1335 (44)	1674 (56)	< 0.001	1.96	1.43–2.70
Other races/ethnities	72 (28)	187 (72)		1.0 (ref)	
* U.S. census region*					
Midwest	902 (42)	1257 (58)	< 0.001	1.41	0.89–2.22
South	315 (46)	375 (54)		1.81	1.12–2.92
West	154 (53)	138 (47)		2.56	1.52–4.29
Northeast	33 (30)	78 (70)		1.0 (ref)	
**First time firing a rifle/shotgun < 10 years old**^e^					
*Sex*					
Male	788 (54)	680 (46)	< 0.001	1.84	1.57–2.16
Female	448 (38)	721 (62)		1.0 (ref)	
* Age*					
16–18	801 (45)	967 (55)	0.028	0.83	0.70–0.99
13–15 years	441 (50)	444 (50)		1.0 (ref)	
*Residence*					
Farm	656 (51)	625 (49)	< 0.001	2.12	1.68–2.67
Country/not farm	431 (48)	476 (53)		1.75	1.37–2.23
Town	155 (33)	312 (67)		1.0 (ref)	
* Race/ethnicity*					
Non-Hispanic White	1171 (47)	1310 (53)	0.215	0.72	0.98–1.39
Other races/ethnicities	71 (42)	97 (58)		1.0 (ref)	
*U.S. census region*					
Midwest	741 (42)	1014 (58)	< 0.001	1.62	0.97–2.69
South	334 (59)	231 (41)		3.32	1.96–5.63
West	139 (57)	103 (43)		3.08	1.75–5.42
Northeast	24 (31)	54 (69)		1.0 (ref)	

^a^The analyses performed controlled for all other listed variables in the models

^b^The sum of n for a variable may not equal the total Group N due to missing values

^c^The total number of cases used in the logistic regression model was 3199

^d^The total number of cases used in the logistic regression model was 3199

^e^The total number of cases used in the logistic regression model was 2607

With respect to having fired a rifle/shotgun, multivariable logistic regression analysis revealed that males had 4.5 times greater odds than females. Individuals who lived on a farm or in the country/not on a farm had 3.4 and 2.4 times greater odds, respectively, than participants who lived in town. NH Whites had 2.5 times greater odds than other races/ethnicities; and FFA members from the Midwest, South and West had 2.0, 2.4 and 3.2 times higher odds, respectively, than those in the Northeast.

Among those who had fired rifles/shotguns frequently (defined as > 100 times) males, those living on a farm, NH Whites, and individuals from the West had the highest percentages. Males had odds over 5 times higher than females of frequent use. Participants who lived on farms and in the country/not on a farm had 2.8 and 1.9 times greater odds, respectively, than those from town. The odds of NH Whites having fired a rifle/shotgun > 100 times was about twice that of other races/ethnicities. Individuals living in the Midwest, South and West regions had 1.4, 1.8 and 2.6 times greater odds, respectively than those from the Northeast.

As compared to their peers, males, younger teenagers, those living on farms, and respondents from the South and West had significantly higher percentages that had fired a rifle/shotgun for the first time before 10 years of age. Multivariable regression analysis demonstrated males having odds nearly twice that of females for having fired a rifle/shotgun before 10 years of age. The odds of first firing a rifle/shotgun before 10 years of age for those living on a farm or in the country/not a farm were 2.1 and 1.8 times higher, respectively, than those living in town. Participants from the West and the South had 3.1 and 3.3 times greater odds, respectively, as compared to those from the Northeast.

### Comparisons of handgun use

Over two-fifths (956/2168, 44%) of participants had fired a handgun before they were 11 years old and more than two thirds (1463/2168, 67%) had done so before the age of 13. The mean age members had first fired a handgun was 11.1 years (SD 3.0 years).

Males, NH Whites, and those living on farms or in the country/not on a farm, all had greater percentages who had fired a handgun relative to their peers. See Table 3. Participants from the West and South had significantly higher proportions that had fired a handgun than those from the Midwest with the lowest proportion having fired a handgun from the Northeast.

**Table 3:** Demographic comparisons of handgun use among survey respondents at the 2021 National FFA Convention & Expo

Variables	Cross tab analysis			Logistic regression analysis^a^	
	Yes	No	*p* value	OR	95% CI
	n (Row %)^b^	n (Row %)^b^			
**Fired a handgun**^c^					
* Sex*					
Male	1320 (82)	281 (18)	< 0.001	3.72	3.15–4.39
Female	903 (56)	717 (44)		1.0 (ref)	
*Age*					
16–18 years	1498 (69)	667 (31)	0.477	1.11	0.94–1.32
13–15 years	734 (68)	346 (32)		1.0 (ref)	
*Residence*					
Farm	1080 (73)	395 (27)	< 0.001	1.80	1.46–2.21
Country/not farm	765 (69)	337 (31)		1.55	1.25–1.92
Town	393 (59)	278 (41)		1.0 (ref)	
*Race/ethnicity*					
Non-Hispanic White	2077 (70)	911 (30)	0.008	1.39	1.03–1.85
Other races/ethnicities	158 (61)	99 (39)		1.0 (ref)	
* U.S. census region*					
Midwest	1435 (67)	709 (33)	< 0.001	2.06	1.36–3.11
South	509 (74)	176 (26)		3.13	2.01–4.87
West	229 (78)	63 (22)		3.97	2.41–6.55
Northeast	52 (48)	57 (52)		1.0 (ref)	
**Frequent firing of handguns (defined as > 100 times)**^d^					
*Sex*					
Male	566 (35)	1035 (65)	< 0.001	4.78	3.94–5.81
Female	165 (10)	1455 (90)		1.0 (ref)	
*Age*					
16–18 years	503 (23)	1662 (77)	0.288	1.15	0.96–1.39
13–15 years	233 (22)	847 (78)		1.0 (ref)	
*Residence*					
Farm	380 (26)	1095 (74)	< 0.001	1.91	1.47–2.48
Country/not farm	260 (24)	842 (76)		1.79	1.36–2.35
Town	95 (14)	576 (86)		1.0 (ref)	
*Race/ethnicity*					
Non-Hispanic White	685 (23)	2303 (77)	0.118	1.28	0.90–1.85
Other races/ethnicities	48 (19)	209 (81)		1.0 (ref)	
*U.S. census region*					
Midwest	442 (21)	1702 (79)	< 0.001	1.80	0.95–3.38
South	181 (26)	504 (74)		2.62	1.37–5.02
West	98 (34)	194 (66)		3.65	1.86–7.18
Northeast	12 (11)	97 (89)		1.0 (ref)	
**First time firing a handgun < 12 years old**^e^					
*Sex*					
Male	701 (54)	601 (46)	< 0.001	1.37	1.15–1.64
Female	385 (45)	468 (55)		1.0 (ref)	
*Age*					
16–18	669 (46)	782 (54)	< 0.001	0.57	0.47–0.69
13–15 years	423 (59)	289 (41)		1.0 (ref)	
*Residence*					
Farm	537 (51)	508 (49)	0.426	1.14	0.90–1.45
Country	375 (51)	364 (49)		1.07	0.83–1.38
Town	182 (48)	201 (52)		1.0 (ref)	
* Race/ethnicity*					
Non-Hispanic White	78 (52)	73 (48)	0.766	1.10	0.78–1.56
Other races/ethnicities	1014 (50)	998 (50)		1.0 (ref)	
*U.S. census region*					
Midwest	662 (48)	730 (52)	< 0.001	1.92	1.03–3.57
South	285 (58)	206 (42)		3.08	1.63–5.82
West	128 (58)	94 (42)		3.06	1.57–5.98
Northeast	16 (31)	35 (69)		1.0 (ref)	

^a^The analyses performed controlled for all other listed variables in the models

^b^The sum of n for a variable may not equal the total Group N due to missing values

^c^The total number of cases used in the logistic regression model was 3178

^d^The total number of cases used in the logistic regression model was 3178

^e^The total number of cases used in the logistic regression model was 2132

Multivariable logistic regression analysis showed males had odds 3.7 times greater than females of having fired a handgun. Individuals living on a farm and those from the country/not a farm had odds 1.8 and 1.6 times higher, respectively, than those living in town, and NH Whites had odds 1.3 times greater than other races/ethnicities. The odds of having fired a handgun were 2.1, 3.1 and 4.0 times greater for those from the Midwest, South and West, respectively, as compared to the Northeast.

Groups with higher percentages of more frequent use of handguns as compared to their peers included males, those who lived on farms or in the country/not on a farm, and those from the South and West. The odds of males reporting firing a handgun > 100 times was nearly 5 times greater than females. Participants living on a farm or in the country/not on a farm had odds of frequent use that were 1.9 and 1.8 times higher, respectively, than those from towns. Members from the South and the West had 2.6 and 3.7 times greater odds, respectively, of frequent use that those from the Northeast.

With regards to having first fired a handgun before 12 years of age, males and younger teenagers had higher proportions as compared to females and older teenagers. Those from the South and West had higher percentages than the Midwest, who subsequently had greater percentages than the Northeast. Multivariable logistic regression analysis showed males had odds 1.4 times greater than females and younger teens had odds 1.8 times greater than older teens of having first fired a handgun at < 12 years of age. Those from the Midwest, West and South had odds that were 1.9, 3.1 and 3.1 times higher, respectively, than members from the Northeast.

### Hunting

A greater proportion of males had hunted as compared to females. See Table 4**.** Those from farms or from the country/not on a farm, NH Whites and participants from the South all had greater proportions that reported hunting relative to their counterparts. Males had odds 2.9 times greater than females of having gone hunting. Those from farms had 3.0 times and those from the country/not a farm had 2.3 times the odds of having gone hunting as compared to participants from towns. The odds of having gone hunting were 1.9 times higher for both NH Whites versus other races/ethnicities and those from the South versus the Northeast.

**Table 4:** Demographic comparisons of hunting behaviors among survey respondents at the 2021 National FFA Convention & Expo

Variables	Cross tab analysis			Logistic regression analysis^a^	
	Yes	No	*p* value	OR	95% CI
	n (Row %)^b^	n (Row %)^b^			
**Had gone hunting**^c^					
*Sex*					
Male	1229 (77)	377 (23)	< 0.001	2.90	2.47–3.39
Female	848 (53)	765 (47)		1.0 (ref)	
* Age*					
16–18 years	1391 (64)	770 (36)	0.966	1.06	0.90–1.25
13–15 years	695 (64)	386 (36)		1.0 (ref)	
*Residence*					
Farm	1066 (72)	408 (28)	< 0.001	3.01	2.46–3.68
Country/not farm	722 (66)	379 (34)		2.24	1.82–2.76
Town	300 (45)	369 (55)		1.0 (ref)	
*Race/ethnicity*					
Non-Hispanic White	1954 (65)	1030 (35)	< 0.001	1.89	1.41–2.50
Other races/ethnicites	128 (50)	129 (50)		1.0 (ref)	
* U.S. census region*					
Midwest	1335 (62)	808 (38)	< 0.001	1.08	0.71–1.63
South	488 (71)	195 (29)		1.85	1.19–2.87
West	191 (66)	99 (34)		1.40	0.87–2.27
Northwest	64 (58)	46 (42)		1.0 (ref)	
**Went hunting the first time when < 10 years old**^d^					
* Sex*					
Male	498 (41)	721 (59)	< 0.001	1.34	1.11–1.62
Female	275 (33)	561 (67)		1.0 (ref)	
*Age*					
16–18 years	493 (36)	885 (64)	0.022	0.79	0.65–0.96
13–15 years	281 (41)	405 (59)		1.0 (ref)	
* Residence*					
Farm	419 (40)	632 (60)	0.002	1.75	1.31–2.34
Country/not farm	272 (38)	444 (62)		1.48	1.09–2.01
Town	85 (29)	213 (71)		1.0 (ref)	
*Race/ethnicity*					
Non-Hispanic White	728 (38)	1203 (62)	0.690	1.16	0.78–1.75
Other races/ethnicities	46 (36)	82 (64)		1.0 (ref)	
*U.S. census region*					
Midwest	449 (34)	872 (66)	< 0.001	3.38	1.59–7.19
South	273 (57)	206 (43)		9.07	4.21–19.55
West	43 (23)	148 (77)		1.90	0.84–4.32
Northeast	9 (14)	55 (86)		1.0 (ref)	

^a^The analyses performed controlled for all other listed variables in the models

^b^The sum of n for a variable may not equal the total Group N due to missing values

^c^The total number of cases used in the logistic regression model was 3175

^d^Includes only those that had gone hunting. The total number of cases used in the logistic regression model was 2034

Nearly one third (667/2066, 32%) of those that went hunting stated they first did so at < 9 years old, 55% (1142/2066) at < 11 years old, and 76% (1575/2066) < 13 years of age. Of those who had gone hunting with a rifle/shotgun, the average age they first participated was 10.2 years (SD 3.0).

Males, younger teenagers, and those from farms and from the country/not a farm had higher percentages as compared to their peers of having first hunted at < 10 years old. The South and Midwest both had higher percentages as compared to the West and Northeast. Males and younger teens had odds 1.3 times greater than females and older teens, respectively, of having first gone hunting at < 10 years of age. Those from farms had 1.8 times and those from the country/not a farm had 1.5 times higher odds of having first gone hunting at < 10 years than participants from towns. The odds of having first gone hunting at < 10 years was 3.4 and 9.1 times greater for those from the Midwest and South, respectively, as compared to those from the Northeast.

### School or club shooting teams

Those with higher proportions reporting having been a shooting team member included males as compared to females, and those living on farms or in the country/not a farm as compared to those from towns. See Table 5. The Midwest, South and West had higher percentages as compared to the Northeast. The odds of being or having been a shooting team member was 2.5 times greater for males as compared to females, 1.9 times greater for those from farms as compared to towns, and 1.4 times greater for those from the county/not a farm as compared to towns. The South, West and Midwest all had odds greater than twice that of those from the Northeast of being or having been a member of a shooting team.

**Table 5:** Demographic comparisons related to membership in a school or club shooting team among survey respondents at the 2021 National FFA Convention & Expo

Variables	Cross tab analysis			Logistic regression analysis^a^	
	Yes	No	*p* value	OR	CI
	n (Row %)^b^	n (Row %)^b^			
**Member of a school or club shooting team**					
Sex					
Male	489 (31)	1094 (69)	< 0.001	2.52	2.11–3.01
Female	234 (15)	1370 (85)		1.0 (ref)	
*Age*					
16–18 years	484 (23)	1656 (77)	0.927	1.03	0.86–1.23
13–15 years	244 (23)	828 (77)		1.0 (ref)	
*Residence*					
Farm	404 (28)	1062 (72)	< 0.001	1.94	1.52–2.50
Country/not farm	224 (21)	863 (79)		1.37	1.05–1.78
Town	100 (15)	562 (85)		1.0 (ref)	
*Race/ethnicity*					
Non-Hispanic White	680 (23)	2280 (77)	1.250	1.05	0.87–1.80
Other races/ethnicities	48 (19)	203 (81)		1.0 (ref)	
*U.S. census region*					
Midwest	501 (24)	1617 (76)	0.010	2.39	1.26–4.53
South	149 (22)	529 (78)		2.27	1.17–4.38
West	63 (22)	227 (78)		2.17	1.08–4.34
Northeast	11 (10)	98 (90)		1.0 (ref)	

^a^The analysis performed controlled for all other listed variables in the model. The n = 3142 for the analysis

^b^The sum of n for a variable may not equal the total Group N due to missing values

### Firearm safety education

Of adolescents that had taken a certified hunter or firearm safety course, 12% (228/1920) took the course at < 10 years, 33% (640/1920) at 11 or 12 years, 27% (520/1920) at 13 or 14 years, and 28% (532/1920) when ≥ 15 years of age. The average age they completed safety training was 11.9 years (SD 2.4).

Demographic groups with higher proportions that reported taking a firearm safety course included males, those living on farms or in the country/not on a farm, NH Whites, and those living in the West. See Table 6. The odds of having taken a firearm safety course was 1.6 times greater for males as compared to females, 1.3 times greater for older teens as compared to younger teens, and 1.6 and 1.3 times greater for those from farms and from the country/not a farm, respectively, as compared to those from towns.

**Table 6:** Demographic comparisons related to firearm safety training among survey respondents at the 2021 National FFA Convention & Expo

Variables	Cross tab analysis			Logistic regression analysis^a^	
	Yes	No	*p* value	OR	CI
	n (Row %)^b^	n (Row %)^b^			
**Completed firearm safety course**					
*Sex*					
Male	1154 (72)	455 (28)	< 0.001	1.61	1.35–1.92
Female	777 (48)	848 (52)		1.0 (ref)	
*Age*					
16–18 years	1313 (60)	861 (40)	0.168	1.28	1.06–1.53
13–15 years	628 (58)	457 (42)		1.0 (ref)	
*Residence*					
Farm	1001 (67)	483 (33)	< 0.001	1.64	1.29–2.08
Country/not farm	651 (59)	455 (41)		1.28	1.00–1.63
Town	292 (44)	379 (56)		1.0 (ref)	
*Race/ethnicity*					
Non-Hispanic White	1821 (61)	1185 (39)	< 0.001	1.10	0.78–1.56
Other races/ethnicities	119 (47)	134 (53)		1.0 (ref)	
*U.S. census region*					
Midwest	1265 (59)	884 (41)	0.025	0.78	0.48–1.28
South	418 (60)	274 (40)		0.84	0.50–1.41
West	196 (67)	96 (33)		1.15	0.66–2.03
Northeast	58 (53)	51 (47)		1.0 ref)	
*School/club shooting team member*					
Yes	630 (87)	95 (13)	< 0.001	4.20	3.25–5.43
No	1279 (52)	1199 (48)		1.0 (ref)	
* Firearm usage*					
Never Used a Firearm	35 (8)	401 (92)	< 0.001	0.14	0.09–0.21
Both rifle and handgun	1583 (72)	604 (28)		1.80	1.43–2.25
Rifle or handgun only	309 (50)	305 (50)		1.0 (ref)	

^a^The analysis performed controlled for all other listed variables in the model

^b^The sum of n for a variable may not equal the total Group N due to missing values

A higher percentage of participants who had belonged to a school or club shooting team had completed a firearm safety course as compared to respondents who had not, and shooting team members had a 4.2 times greater odds of having taken a course than their peers. Only half of those that had fired a rifle/shotgun or a handgun (but not both) had taken a formal firearm safety course, while nearly three-quarters that had fired both rifles/shotguns and handguns had completed a course. Those who had used both a rifle/shotgun and a handgun had 1.8 times higher odds of having completed a firearm safety course as compared to those who had used a rifle/shotgun or a handgun only.

## Discussion

Investigations regarding the use of firearms by adolescents are generally lacking, making this study a valuable addition to the current literature. Our study of a national sampling of adolescent FFA members found that a marked majority had fired both rifles/shotguns and handguns, and that many used them frequently. We also found that rural children are using firearms at very young ages. When comparing the U.S. census regions, study participants who resided in the Northeast had the least experience with firearms, whereas, respondents from the West and the South had used rifle/shotguns and handguns more frequently and at younger ages. Of the FFA members who had used a firearm, only around two-thirds had taken a certified firearm safety course.

More than one-fifth of participants had been a member of a school or club shooting team. According to the Scholastic Shooting Sports Foundation, youth participation in shooting events in 2015 had increased by 146% in 6 years (National Shooting Sports Foundation 2016). Additionally, the USA High School Clay Target League grew in membership from 1700 to over 43,000 adolescents from 2012 to 2022 (Karp 2018). The League reports that it is the “fastest growing high-school extracurricular activity in the country” (USA High School Clay Target League 2024).

Hunting was also common among our study population across all regions and the majority had done so before 11 years of age. The U.S. Fish and Wildlife Service estimated 14.4 million people hunted in 2022 with 500,000 being 16 and 17 years old—an increase of 66% since 2016 (U.S. Department of the Interior et al. 2016; U.S. Department of the Interior and U.S. Fish and Wildlife Service 2022). There are 29 states that allow children at any age to hunt if supervised by an adult and 6 states that have no age limit specified to hunt unsupervised (Outdoor Empire 2023). A national study of unintentional firearm deaths in children 0–14 years found 11% were hunting-related (Hemenway and Solnick 2015).

Firearm use varied significantly by where participants lived being more common in rural areas, and safety training was more common among rural youth. This is similar to our study of Iowa FFA members (Jennissen et al. 2021), and is consistent with other studies investigating urban and rural differences (Branas et al. 2004; Herrin et al. 2018). Study adolescents that lived outside of towns also had higher percentages that had gone hunting which may help explain their higher proportions that received training as it is often required to obtain a hunting license (Huntin’ Fool 2024).

Our study showed regional patterns of more frequent and earlier firearm use by youth that had some similarities to regional patterns seen for firearm-related injuries. For example, the risk of self-inflicted firearm hospitalization in those < 21 years of age has been highest in the South, followed by the West and then the Midwest, with all three having significantly greater risk odds than the Northeast (McLoughlin et al. 2019). Another study found that for those 0–17 years, Southern states and parts of the Midwest had higher rates for firearm homicides and some of the highest firearm suicide rates were in Western states (Fowler et al. 2017).

Participants in our study reported very young ages at which they first used firearms (means of < 9 ½ years for rifles/shotguns and ~ 11 years for handguns). Given the extremely young ages many rural children in the study were allowed to use firearms, it is likely that their cognitive and physical development were inadequate to assure safe firearm use.

## Limitations

Our study consisted primarily of a rural NH White population. Thus, our findings may not be generalizable to urban areas and populations with more racial and ethnic diversity. Another limitation was that the specific type of firearm safety training participants had received (e.g., in-person vs. online) was not asked in the survey. Additionally, all collected information was self-reported and may be subject to recall bias and social desirability. Surveys were performed independently and anonymously which should have decreased the social desirability effect.

## Conclusions

A large majority of participating FFA members had fired both handguns and rifles/shotguns, many at very young ages. Significant differences in firearm use were noted by demographic factors including the youth’s home setting and their U.S. census region. Nearly a third that had used a firearm had not received formal training. Legislation that requires firearm safety training certification prior to firearm use by youth would help decrease this proportion. Parents and caregivers should receive targeted education regarding firearm safety including advisement when it is developmentally appropriate to introduce youth to firearms and on the importance of safe storage, professional training and the direct supervision of youth when using firearms.

## Data Availability

Data and materials are available to other parties for research purposes after a data sharing agreement plan is agreed to and signed. Those interested should contact the corresponding author.

## Abbreviations

CDC: Centers for Disease Control and Prevention
e.g.: Exempli gratia (for example)
FFA: Formerly stood for Future Farmers of America
TM: Trademark
U.S.: United States
